# Milk cholesterol concentration in mice is not affected by high cholesterol diet- or genetically-induced hypercholesterolaemia

**DOI:** 10.1038/s41598-018-27115-8

**Published:** 2018-06-11

**Authors:** Lidiya G. Dimova, Mirjam A. M. Lohuis, Vincent W. Bloks, Uwe J. F. Tietge, Henkjan J. Verkade

**Affiliations:** Department of Pediatrics, Molecular Metabolism and Nutrition, University of Groningen, University Medical Center Groningen, Groningen, The Netherlands

## Abstract

Breast milk cholesterol content may imply to affect short- and long-term cholesterol homeostasis in the offspring. However, mechanisms of regulating milk cholesterol concentration are only partly understood. We used different mouse models to assess the impact of high cholesterol diet (HC)- or genetically-induced hypercholesterolaemia on milk cholesterol content. At day 14 postpartum we determined milk, plasma and tissue lipids in wild type (WT), LDL receptor knockout (*Ldlr*−/−), and ATP-binding cassette transporter G8 knockout (*Abcg8*−/−) mice fed either low- or 0.5% HC diet. In chow-fed mice, plasma cholesterol was higher in *Ldlr*−/− dams compared to WT. HC-feeding increased plasma cholesterol in all three models compared to chow diet. Despite the up to 5-fold change in plasma cholesterol concentration, the genetic and dietary conditions did not affect milk cholesterol levels. To detect possible compensatory changes, we quantified *de novo* cholesterol synthesis in mammary gland and liver, which was strongly reduced in the various hypercholesterolaemic conditions. Together, these data suggest that milk cholesterol concentration in mice is not affected by conditions of maternal hypercholesterolaemia and is maintained at stable levels via ABCG8- and LDLR-independent mechanisms. The robustness of milk cholesterol levels might indicate an important physiological function of cholesterol supply to the offspring.

## Introduction

Breast milk contains high levels of cholesterol (0.23–0.39 mmol/L) in contrast to most infant formulas (0–0.10 mmol/L)^1–3^. The relatively high cholesterol concentration in breast milk has been suggested to have a lasting impact on the cholesterol homeostasis of the offspring^1,4^. Breast-fed offspring has high plasma cholesterol levels in early life, but lower plasma cholesterol in adulthood, compared to formula-fed individuals^1,5^. The lower plasma cholesterol concentrations in adulthood may relate to long-term cardio-protective effects of breast milk, in accordance with the metabolic programming hypothesis^1,6^. Additionally, we recently demonstrated that maternal ezetimibe-induced lower dietary cholesterol bioavailability during the lactation period in mice decreases cholesterol absorption in the offspring up to adulthood through decreased intestinal NPC1L1 expression^7^.

The mechanisms involved in the regulation of milk cholesterol concentration are only partly understood. With the recent cardiometabolic disease pandemic, dyslipidaemia and disturbances in lipid homeostasis are becoming increasingly common conditions in pregnant and lactating women^8,9^. Maternal hypercholesterolaemia during gestation has been associated with increased plasma cholesterol in the fetus^10,11^. However, it remains unclear to what extent maternal hypercholesterolaemia, either caused by genetic or dietary factors, impacts cholesterol transport across the mammary gland and affects cholesterol concentration in milk with possible effects in the offspring.

Cholesterol in milk can originate from different sources. The predominant fraction of cholesterol reaches the milk via plasma^12^: either from preformed stores, from dietary origin or from *de novo* synthesis in either the mammary gland epithelium cells^13,14^ or the liver^12^. The detailed transport route by which cholesterol in the circulation is taken up by the mammary gland has not been identified. There have been reports suggesting an ApoB-mediated uptake of cholesterol-containing lipoproteins^15^. Several receptors for uptake of cholesterol-rich apolipoprotein B-containing lipoproteins are abundantly expressed in the mammary epithelial cells, amongst which LDL-, VLDL- and CD36-receptors^14^. Other lipoproteins found in plasma, like the high-density lipoproteins, may serve as an alternative source for cholesterol uptake since scavenger receptors from the CD36 family are also expressed in the mammary epithelium^16^. In addition, mammary gland epithelial cells express cholesterol efflux transporters, such as ATP-binding cassette (ABC) transporters ABCG5/ABCG8, ABCA1, and ABCG1, whose expressions fluctuate depending on lactation stage^17–19^ and could possibly impact cholesterol levels in the milk.

We aimed to address the relationship between maternal hypercholesterolaemia and milk cholesterol concentration in mouse models. We analysed milk cholesterol concentrations in lactating mice with hypercholesterolaemia of different severity, induced by dietary and/or genetic manipulations. The dietary means to manipulate plasma cholesterol concentrations consisted of feeding a high-cholesterol diet (0.5% w/w), while genetic manipulation involved the ablation of either the *Abcg8* or the *Ldlr* gene. The ABC cassette G8 protein is a cholesterol transporter primarily expressed on the apical membrane of hepatocytes and enterocytes, where it facilitates export of cholesterol^20^. Interestingly, *Abcg8* is also moderately expressed in the lactating bovine mammary gland and in the murine mammary gland, as demonstrated in literature and online databases^17,19,21^. The LDL-receptor is the dominant transport protein involved in the uptake of apoB100-containing lipoproteins from the plasma^22^, and highly expressed in murine mammary gland^21^. Humans with genetic loss of LDLR function have a severe hypercholesterolaemia that is further increased upon dietary cholesterol exposure^23^. We assessed the potential relevance of cholesterol secretion into milk via the ABCG8 transporter and via mammary gland uptake of cholesterol via the LDL receptor. To assess possible variation in the origin of milk cholesterol in the different models of hypercholesterolaemia, we measured *de novo* cholesterol synthesis in the liver and mammary gland, using deuterated water methodology.

## Results

### High-cholesterol diet increases plasma and hepatic cholesterol levels

**Figure 1:**
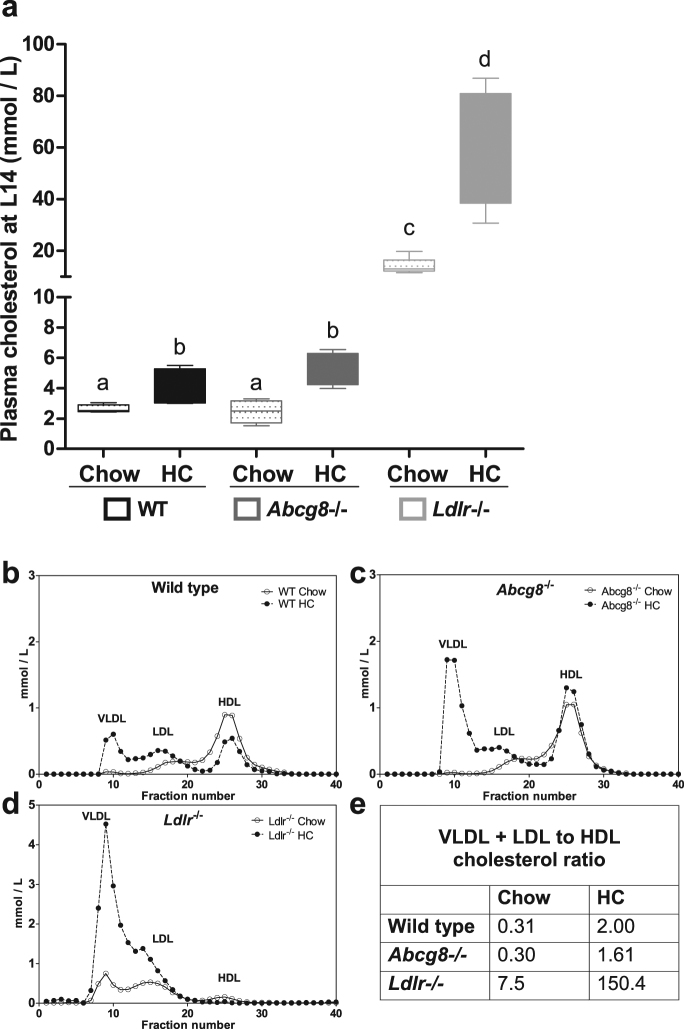
Plasma lipids. (**a**) Total plasma cholesterol levels were measured in whole plasma using a commercially available enzymatic assay (WT Chow, n = 5; WT HC, n = 5; *Abcg8* −/− Chow, n = 4; *Abcg8* −/− HC, n = 5; *Ldlr* −/− Chow, n = 8; *Ldlr* −/− HC, n = 5). Data are presented as median and interquartile range (Tukey). Statistical significance was tested with Kruskal-Wallis post-hoc Conover-Inman; non-different groups share a letter. The threshold of significance was p < 0.05. (**b**–**d**) Cholesterol in lipoprotein fractions following separation by FPLC of pooled plasma samples and (**e**) VLDL + LDL to HDL cholesterol ratios calculated from these results (WT Chow, n = 5; WT HC, n = 5; *Abcg8* −/− Chow, n = 4; *Abcg8* −/− HC, n = 5; *Ldlr* −/− Chow, n = 4; *Ldlr* −/− HC, n = 4). □: low cholesterol diet (Chow); ■: high cholesterol diet (HC).

**Figure 2:**
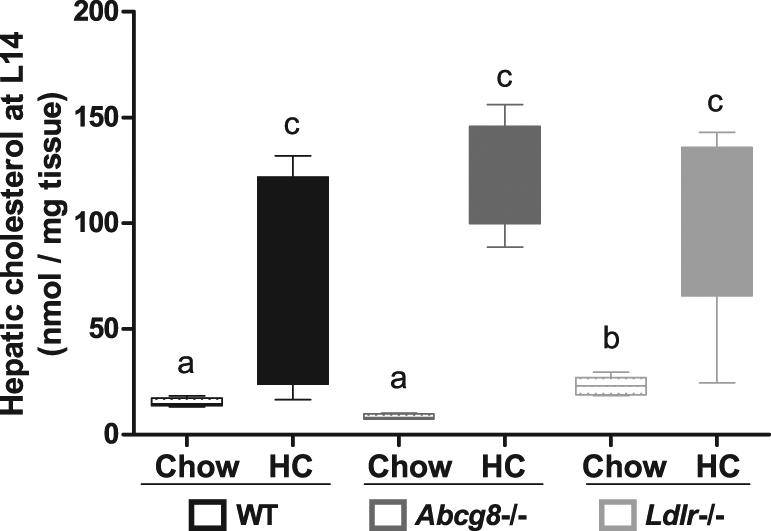
Hepatic cholesterol levels. Hepatic lipids were extracted according to Bligh & Dyer and measured by gas chromatography (WT Chow, n = 5; WT HC, n = 5; *Abcg8* −/− Chow, n = 4; *Abcg8* −/− HC, n = 5; *Ldlr* −/− Chow, n = 8; *Ldlr* −/− HC, n = 5). Data are presented as median and interquartile range (Tukey). □: low cholesterol diet (Chow); ■: high cholesterol diet (HC). Statistical significance was tested with Kruskal-Wallis post-hoc Conover-Inman; non-different groups share a letter. The threshold of significance was p < 0.05.

To assess the isolated effect of ABCG8- or LDLR-deficiency we first measured cholesterol levels in plasma of dams on a chow diet. While ABCG8-deficiency did not affect basal plasma cholesterol, the LDLR-deficient dams displayed marked hypercholesterolaemia (5.2-fold change, p < 0.01, Fig. 1a), mostly due to increased cholesterol levels in LDL and VLDL (Fig. 1b–d). Feeding the dams high cholesterol (HC) diet increased the levels of total plasma cholesterol in all models (Fig. 1a). The size of the effect reached maximum in the *Ldlr*−/− mice (4.8-fold change, p < 0.01) followed by *Abcg8*−/− (2-fold change, p < 0.05) and wild-type (1.5-fold change, p < 0.05). On chow diet, hepatic cholesterol concentration corresponded with the differences in the plasma cholesterol levels: similar levels in wild-type and *Abcg8* knockout mice and 0.6-fold higher in LDLR-deficient mice (p < 0.01). The HC diet increased the cholesterol accumulation in the hepatic tissues of all dams (p < 0.05, Fig. 2). On the HC diet, however, the hepatic cholesterol concentrations did not differ significantly between the three models.

### Milk cholesterol levels are independent of plasma, liver and mammary gland cholesterol levels

**Figure 3:**
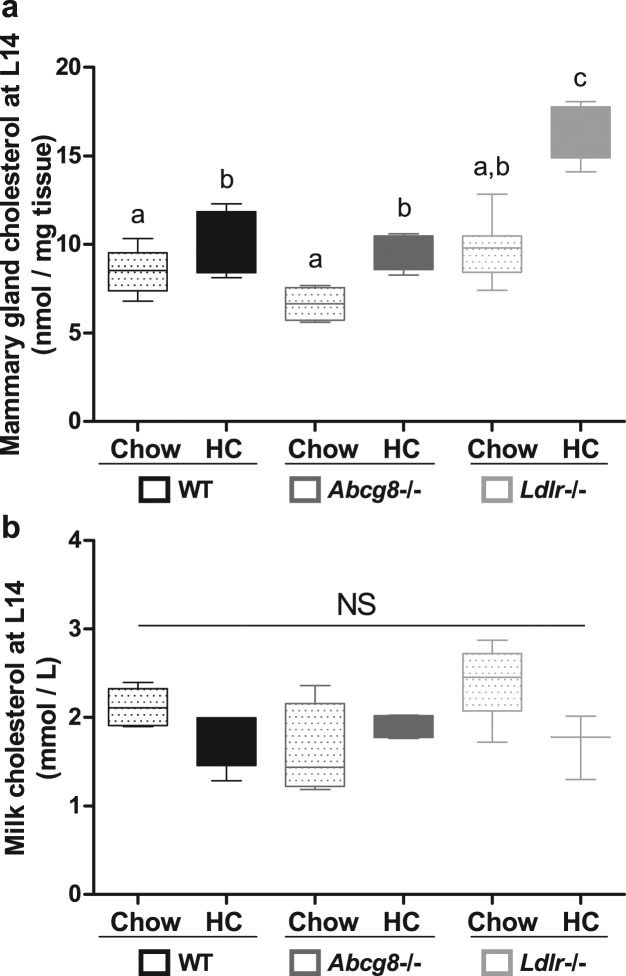
Mammary gland and milk cholesterol. (**a**) The lipid content of mammary tissue was extracted according to Bligh & Dyer and measured by gas chromatography (WT Chow, n = 5; WT HC, n = 5; *Abcg8* −/− Chow, n = 4; *Abcg8* −/− HC, n = 5; *Ldlr* −/− Chow, n = 8; *Ldlr* −/− HC, n = 5). (**b**) Milk samples were obtained after i.p. injection with 1 IU oxytocin by using a modified electric human breast pump. Milk lipids were extracted according to Bligh & Dyer and cholesterol was quantified by gas chromatography (WT Chow, n = 5; WT HC, n = 5; *Abcg8* −/− Chow, n = 4; *Abcg8* −/− HC, n = 4; *Ldlr* −/− Chow, n = 6; *Ldlr* −/− HC, n = 3). Data are presented as median and interquartile range (Tukey). □: low cholesterol diet (Chow); ■: high cholesterol diet (HC). Statistical significance was assessed with Kruskal-Wallis post-hoc Conover-Inman test; non-different groups share a letter. The threshold of significance was p < 0.05.

We then determined whether the hypercholesterolaemia was associated with increased cholesterol content of the mammary glands. On chow diet there were no differences in mammary cholesterol content between genotypes, despite the significantly increased plasma cholesterol levels in *Ldlr*−/− mice (Fig. 3a). The HC diet did not increase mammary cholesterol content in the WT mice, in contrast to the *Abcg8* and *Ldlr* knockout mice (+39%, p < 0.05; and +62%, p < 0.01 respectively; Fig. 3a). Interestingly, the HC diet-induced hypercholesterolaemia did not affect the milk cholesterol concentrations in any of the three models, with milk cholesterol levels ranging between 1.7–2.3 mM (interquartile range) (Fig. 3b).

In order to analyse the possible association between milk cholesterol levels and plasma and mammary gland cholesterol levels and nest size, we performed regression analysis. Nest sizes (range: 2–8 pups) were not correlated with milk cholesterol levels. Cholesterol levels in mammary gland tissue were strongly and positively related to plasma cholesterol levels in all three models (WT r^2^ = 0.54, p = 0.016; *Abcg8*−/− r^2^ = 0.61, p = 0.013; *Ldlr*−/− r^2^ = 0.51, p = 0.0096). In none of the three groups were plasma and milk cholesterol levels significantly correlated. Ratios of VLDL + LDL to HDL cholesterol as calculated from FPLC fractions (Fig. 1e) were also unrelated to milk cholesterol levels.

### *De novo* cholesterol synthesis is strongly decreased in high cholesterol-fed mice

**Figure 4:**
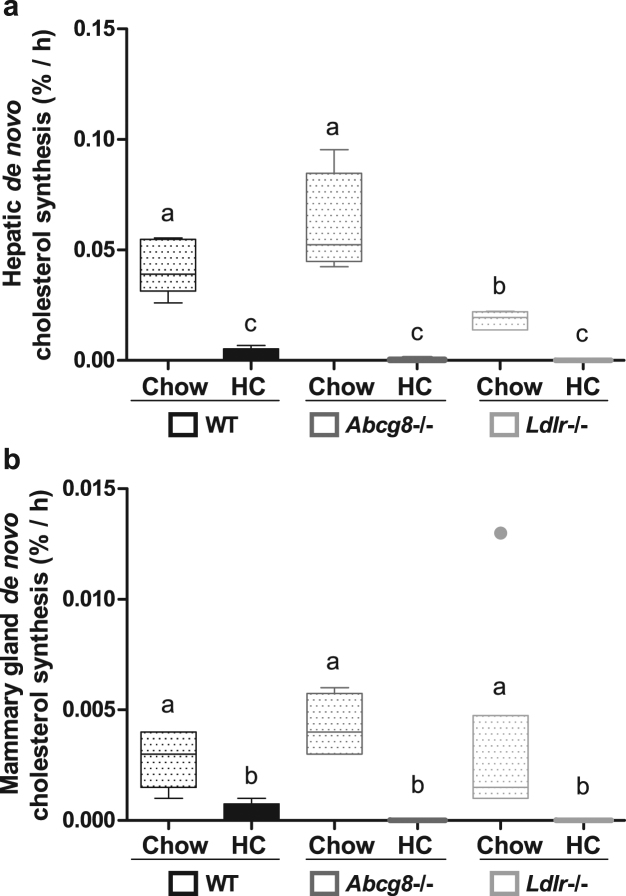
*De novo* cholesterol synthesis. On L14 the dams received deuterium water i.p. one hour before harvesting the organs. The mammary gland was milked 10 minutes before harvesting. The fraction of deuterium-incorporated cholesterol in liver and mammary gland was assessed using isotope ratio mass spectrometry (IRMS). (**a**) De novo cholesterol synthesis in the liver (%/h). (**b**) De novo cholesterol synthesis in the mammary gland (%/h). (WT Chow, n = 5; WT HC, n = 4; *Abcg8* −/− Chow, n = 4; *Abcg8* −/− HC, n = 5; *Ldlr* −/− Chow, n = 6; *Ldlr* −/− HC, n = 4). Data are presented as median and interquartile range (Tukey). Statistical significance was tested with Kruskal-Wallis post-hoc Conover-Inman; non-different groups share a letter. □: low cholesterol diet (Chow); ■: high cholesterol diet (HC). The threshold of significance was p < 0.05.

The increased plasma, hepatic and mammary gland cholesterol levels in the hypercholesterolaemic models did not translate into increased milk cholesterol concentrations. We then tested the possibility that the stable concentrations were obtained by suppression of systemic or local cholesterol synthesis. In all chow-fed groups there was *de novo* hepatic and mammary gland cholesterol synthesis (Fig. 4). Feeding the HC diet strongly reduced the cholesterol synthesis rate in liver (Fig. 4a) and mammary gland (Fig. 4b) in all three models.

Next, we used linear regression analysis to assess the possible relationship between the *de novo* synthesis in mammary gland and the milk cholesterol concentration. The milk cholesterol levels did not correlate with the fraction of *de novo* synthesized cholesterol in mammary gland in any of the three groups (WT r^2^ = 0.03, p = 0.66; *Abcg8*−/− r^2^ = 0.07, p = 0.51; *Ldlr*−/− r^2^ = 0.05, p = 0.64).

## Discussion

We addressed the relationship between maternal hypercholesterolaemia, induced by dietary or genetic means, and milk cholesterol concentrations in mice. Our data demonstrate that milk cholesterol concentration is not affected by induction of severe hypercholesterolaemia and increased cholesterol levels in liver and mammary gland. Clearly, the ABC-cassette transporter ABCG8 and the LDL receptor do not have a critical role in defining milk cholesterol concentration, since their inactivation did not change it. Our data demonstrate the apparent robustness of milk cholesterol levels, which could support important physiological functions for the offspring.

The milk cholesterol concentration was not affected by genetic inactivation of two candidate genes with a possible role in cholesterol transport towards milk, nor by high cholesterol diet-induced hypercholesterolaemia. This observation indicates that either the gene products are not involved, or that alternative transporting mechanisms ensure redundancy in the supply of cholesterol destined for secretion into the milk. The hypothesis that the LDL receptor is involved in milk cholesterol transport was based on findings describing an association between lactation and increased mammary gland expression of *LDLR* in human subjects^14^ and high LDLR expression in the murine mammary gland^21^. In addition, lactation in rodents is characterized by an increase in circulating LDL^24^, compatible with a role for the low-density lipoproteins as a source for milk cholesterol. Our data indicate that uptake of cholesterol by the mammary gland can be conducted quantitatively by LDLR-independent mechanism(s). We cannot exclude that an alternative, LDLR-independent mechanism involves an alternative receptor for LDL uptake. In support of this notion, radioactivity studies in mice have shown the transfer of ApoB100 across the mammary epithelium towards the milk to take place at the same extent in both wild-type and LDLR-deficient mice^15^. Possibly VLDL and LRP receptors^25^, or even CD36^26^ can substitute for LDLR-deficiency. The hypothesis that ABCG8 is involved in milk cholesterol transport rests on the increased expression levels of the heterodimer ABCG5/ABCG8 in lactating bovine mammary glands^17,19^, and appreciable expression of ABCG8 in murine mammary gland^21^. In hepatocytes and intestinal epithelial cells the ABCG5/ABCG8 dimer is expressed at the apical membrane^20^ where it is essential for the export of free cholesterol towards the bile and intestinal lumen, respectively^27^. Our data, however, does not support a critically important role for ABCG8 in the process of cholesterol efflux across the mammary gland epithelium. The unchanged plasma cholesterol levels in *Abcg8*−/− mice on chow may be related to the fact that the diet used contained no cholesterol. Apparently neither the LDL receptor nor ABCG8 is crucial for cholesterol transport towards milk in our experimental setup. In order to further explore the mechanistic effects of genetic ablation of LDLR and ABCG8 on milk production, additional studies in an *in vitro* model would be helpful. Unfortunately, however, there is no established reliable *in vitro* system for lactating mammary gland cells available to study alveolar mammary gland epithelial cell cholesterol transfer^28^.

*De novo* cholesterol synthesis has been shown to contribute to milk cholesterol^12^. For the dams, cholesterol demand is increased during lactation, corresponding with increased expression of cholesterol synthesis genes in both liver and mammary glands of bovines, rodents and humans^13,14,29^. We found 12-fold higher fractional cholesterol synthesis rates in liver compared to mammary gland, which is in agreement with previous studies demonstrating a larger contribution to milk cholesterol originating from hepatic than from mammary synthesis^12^. The lower mammary gland cholesterol synthesis compared with hepatic synthesis also corresponds to the expression levels of the *Hmgcr* gene in the two tissues, encoding for the rate-limiting enzyme of cholesterol synthesis^13^. In each of the three murine genotypes, dietary cholesterol supplementation strongly decreased *de novo* cholesterol synthesis in liver and in mammary gland, similarly to observations in rats^30^. The decreased *de novo* synthesis rates in liver and mammary gland, however, did not decrease milk cholesterol levels. The cholesterol synthesis rate is apparently not a critical driver for the amount of cholesterol secreted into milk. Rather, it seems that milk cholesterol concentration is robust and “protected” against profound hypercholesterolaemia despite strongly increased tissue cholesterol levels. In addition, comparable to respective compensatory changes in other organs such as the liver, also in the mammary gland cholesterol synthesis decreased in response to dietary cholesterol feeding.

The use of whole-body inactivation of specific genes, as utilized in this study, is comprehensively associated with systemic changes in cholesterol metabolism and apolipoprotein balance. Employing mammary gland-specific genetic models would exclude the influence of hepatic or intestinal deficiency in our mice. However, the present lack of influence on milk cholesterol concentration in whole body-knockouts does not support the possibility that organ-specific inactivation would greatly affect milk cholesterol concentrations.

We would like to hypothesize on the physiological explanation(s) of the present findings. First, it is tempting to speculate that the apparent robustness of the cholesterol concentration in milk relates to physiological importance in milk secretion. The importance of a stable milk cholesterol concentration could relate to the process of secretion of milk lipids, in particular triglycerides. Within the alveolar cells of the mammary gland the secretory lipids are shaped in single phospholipid layer-wrapped lipid droplets. During exocytosis the lipid droplets acquire an additional cholesterol-rich phospholipid bilayer, resulting in the formation of the milk-fat globule (MFG)^31^. Milk cholesterol is mainly present as unesterified cholesterol in the MFG-membrane (85–90%) and the other part as cholesteryl esters in the MFG-core^32,33^. The packaging of the lipid droplets with the MFG membrane, which is essential for their secretion, may therefore translate into a rather stable cholesterol content in milk, based on its role as an emulsion-stabilizing component as part of the MFG-membrane. Second, the robust cholesterol concentration in milk could also underline the hypothesized physiological function of milk cholesterol for later health of the offspring. In contrast to breast milk, the fat globules of common infant milk formula are smaller in size and differ in composition, being coated with milk proteins instead of a phospholipid and cholesterol-rich membrane^34,35^. Indeed, infant formulas hardly contain cholesterol^3^. Cholesterol in early life is not considered an essential dietary component since infants are capable of *de novo* cholesterol synthesis, and thus do not critically depend on milk for their cholesterol supply. As expected, infants fed cholesterol-free formula have increased cholesterol synthesis rates compared to breast-fed infants^36^. Interestingly, however, adult individuals who had been breast-fed as infant have lower total and pro-atherogenic LDL-cholesterol compared to previously formula-fed subjects^3^. This has led to the hypothesis that early life cholesterol supply can program cholesterol homeostasis in later life. In support of this notion, we recently reported indications that dietary cholesterol availability in early life of mice determines the set-point for cholesterol absorption efficiency at adult age^7^. The rather strictly regulated concentration of milk cholesterol found in this study could support the relevance of a stable cholesterol supply for its programming importance. Third, a stable supply of dietary cholesterol could theoretically be relevant for the development of intestinal microbiota in early life. The cholesterol synthesis rate, the biliary cholesterol secretion and the fecal cholesterol excretion have all been shown to correlate with abundance of certain bacterial taxa in hamsters^37^. Additionally, conversion of cholesterol to the neutral sterol coprostanol by the intestinal microbiota is delayed in breast-fed infants^38^, indicating inhibited growth of certain bacterial groups. Yet, a recent study by ourselves in (adult) LDLR-deficient mice does not support the concept that dietary cholesterol has a substantial impact on shaping the intestinal microbiota, since neither the composition nor the functionality of the intestinal microbiota were affected even after prolonged dietary cholesterol exposure^39^.

In conclusion, our results clearly demonstrate that milk cholesterol levels are resistant to maternal high cholesterol diet- and genetically-induced hypercholesterolaemia in mice. We speculate that the robust maintenance of stable milk cholesterol levels may serve relevant physiological functions in the offspring, such as programming of long-term health benefits. Further research however, is required to firmly establish such cause-effect relationships.

## Materials and Methods

### Animal studies

Female C57BL/6J (n = 10), *Ldlr* knockout^40^ (n = 13) and *Abcg8* knockout^20,41^ (n = 9) mice were housed in temperature controlled-conditions with 12:12 light dark cycles and maintained on chow diet (RMH-B, ABDiets, Woerden, Netherlands) with listed specified ingredients: wheat, meat meal, yellow dent corn, whole oats, wheat middlings, alfalfa, soya oil, dried yeast, dicalcium phosphate, calcium carbonate, NaCl, dl-methionine, vitamins and trace elements. Breeding was initiated between 8–12 weeks of age. Due to accumulation of dietary xenosterols, *Abcg8*−/− mice are infertile, which is relieved upon ezetimibe treatment^42^. Therefore, in order to facilitate fertilization, *Abcg8*−/− females were pre-treated for 3 weeks with 0.005% ezetimibe provided via the food, which was removed from the diet once pregnancy was confirmed in accordance with Solca *et al*.^42^. The rest of the models were fed chow until E18, when half of the mice received 0.5% cholesterol diet. Lactation day 1 (L1) was considered the day at which pups were born. On L14 the dams were injected i.p. with 2.3 mL 99% ^2^H_2_O (deuterium oxide) per 100 g BW, containing 0.9% NaCl. After 50 minutes, milk was collected for 10 minutes (details see below) directly followed by termination and harvesting of blood, liver, and mammary glands. All animal experiments were approved by the ethical committee for animal experimentation at the University of Groningen and performed in accordance with relevant guidelines and regulations.

### Milk collection

At lactation day 14^43^, the dams were separated from the pups for 3 hours followed by i.p. injection of 1 IU oxytocin (Synthocinon®, Sigma-Tau Industrie Farmaceutiche Riunite, Rome, Italy). Milk samples were collected continuously for 10 minutes from the mammary gland of isoflurane-anesthetized mouse with the aid of a modified human electric breast pump (Calypso, Ardo Medical AG, Unterägeri, Switzerland). The samples were initially preserved at 4 °C during collection and further stored at −80 °C until use.

### Determination of milk cholesterol

Thawed milk samples were homogenized by continuous vortexing. 25 to 50 µL of milk sample was subjected to lipid extraction according to Bligh & Dyer^44^. Unesterified cholesterol was subsequently derivatized to cholesteryl acetate and quantified by gas chromatography, using 5-alpha cholestane as internal standard^45^.

### Total plasma cholesterol and lipoprotein profiles

Total plasma cholesterol was measured enzymatically using a commercially available kit (Roche Diagnostics GmbH, Mannheim, Germany). Lipoprotein fractions of pooled plasma samples (n = 3–5) were separated via fast protein liquid chromatography gel filtration using a superose 6 column (GE Healthcare, Little Chalfont, UK) as published^46^. Samples were chromatographed at a flow rate of 0.5 ml/min, and lipoprotein fractions of 500 μl each were collected. Individual fractions were assayed for cholesterol concentrations using a commercial kit (Roche Diagnostics GmbH, Mannheim, Germany).

### Hepatic and mammary gland total cholesterol quantification

Liver and mammary gland tissues were homogenized using RNAse free-beads and the TissueLyser LT system (Qiagen GmbH, Hilden, Germany). Lipids were extracted according to Bligh & Dyer^44^. Cholesterol was de-esterified according to Ichihara *et al*.^47^. Free cholesterol underwent acetylation followed by quantification using gas chromatography (GC, Agilent 6890, Amstelveen, the Netherlands)^45^.

### Organ-specific de novo cholesterol synthesis

Liver- and mammary gland-specific *de novo* cholesterol synthesis was quantified using the deuterium incorporation method^48^. Briefly, at L14 the dams were injected i.p. with deuterated water (2.3 mL/100 g BW, 99% ^2^H_2_O, 0.9% NaCl) and terminated after 60 minutes by cardiac puncture^49^. We used a non-injected control mouse for determining the number of hydrogen atoms incorporated in a single newly synthesized cholesterol molecule as measured by GC-MS. Following lipid extraction and de-esterification, the abundance of deuterium-substituted hydrogen atoms was determined by isotope ratio mass spectrometry (IRMS). Synthesis rates were determined as previously published^50^.

### Statistical analysis

The significance of dietary influence within the different genotypes and the analysis of variance between genotypes in the same dietary condition was performed with Kruskal-Wallis followed by a multiple comparisons adjustment using Conover-Inman test. P-values below 0.05 were considered significant.

The datasets generated during and/or analysed during the current study are available from the corresponding author on reasonable request.

## References

[1] Delplanque B, Gibson R, Koletzko B, Lapillonne A, Strandvik B (2015). Lipid Quality in Infant Nutrition: Current Knowledge and Future Opportunities. J. Pediatr. Gastroenterol. Nutr..

[2] Kamelska AM, Pietrzak-Fiećko R, Bryl K (2013). Determination of cholesterol concentration in human milk samples using attenuated total reflectance Fourier transform infrared spectroscopy. J. Appl. Spectrosc..

[3] Wong WW, Hachey DL, Insull W, Opekun AR, Klein PD (1993). Effect of dietary cholesterol on cholesterol synthesis in breast-fed and formula-fed infants. J. Lipid Res..

[4] Mott GE, Jackson EM, McMahan CA, McGill HC (1990). Cholesterol metabolism in adult baboons is influenced by infant diet. J. Nutr..

[5] Owen CG (2008). Does initial breastfeeding lead to lower blood cholesterol in adult life? A quantitative review of the evidence. Am. J. Clin. Nutr..

[6] Barker DJ (1995). The fetal and infant origins of disease. Eur. J. Clin. Invest..

[7] Dimova LG (2017). Inhibiting Cholesterol Absorption During Lactation Programs Future Intestinal Absorption of Cholesterol in Adult Mice. Gastroenterology.

[8] WHO, Obesity and overweight, retrieved from: http://www.who.int/mediacentre/factsheets/fs311/en/ on March 29, 2018.

[9] Napoli C, Infante T, Casamassimi A (2011). Maternal-foetal epigenetic interactions in the beginning of cardiovascular damage. Cardiovasc. Res..

[10] Palinski W (2014). Effect of maternal cardiovascular conditions and risk factors on offspring cardiovascular disease. Circulation.

[11] Palinski W, Napoli C (1999). Pathophysiological events during pregnancy influence the development of atherosclerosis in humans. Trends Cardiovasc. Med..

[12] Long CA, Patton S, McCarthy RD (1980). Origins of the cholesterol in milk. Lipids.

[13] Rudolph MC (2007). Metabolic regulation in the lactating mammary gland: a lipid synthesizing machine. Physiol. Genomics.

[14] Mohammad MA, Haymond MW (2013). Regulation of lipid synthesis genes and milk fat production in human mammary epithelial cells during secretory activation. Am. J. Physiol. Endocrinol. Metab..

[15] Monks J (2001). A lipoprotein-containing particle is transferred from the serum across the mammary epithelium into the milk of lactating mice. J. Lipid Res..

[16] Landschulz KT, Pathak RK, Rigotti A, Krieger M, Hobbs HH (1996). Regulation of scavenger receptor, class B, type I, a high density lipoprotein receptor, in liver and steroidogenic tissues of the rat. J. Clin. Invest..

[17] Farke C, Meyer HH, Bruckmaier RM, Albrecht C (2008). Differential expression of ABC transporters and their regulatory genes during lactation and dry period in bovine mammary tissue. J. Dairy Res..

[18] Mani O (2010). Expression, localization, and functional model of cholesterol transporters in lactating and nonlactating mammary tissues of murine, bovine, and human origin. Am. J. Physiol. Regul. Integr. Comp. Physiol..

[19] Viturro E, Farke C, Meyer HH, Albrecht C (2006). Identification, sequence analysis and mRNA tissue distribution of the bovine sterol transporters ABCG5 and ABCG8. J. Dairy Sci..

[20] Klett EL, Lee MH, Adams DB, Chavin KD, Patel SB (2004). Localization of ABCG5 and ABCG8 proteins in human liver, gall bladder and intestine. BMC Gastroenterol..

[21] Hruz T (2008). Genevestigator v3: a reference expression database for the meta-analysis of transcriptomes. Adv. Bioinformatics.

[22] Marcel YL (1987). Mapping of human apolipoprotein B antigenic determinants. Arteriosclerosis.

[23] Ito MK, Watts GF (2015). Challenges in the Diagnosis and Treatment of Homozygous Familial Hypercholesterolemia. Drugs.

[24] Smith JL (1998). Effect of pregnancy and lactation on lipoprotein and cholesterol metabolism in the rat. J. Lipid Res..

[25] Lillis AP, Van Duyn LB, Murphy-Ullrich JE, Strickland DK (2008). LDL receptor-related protein 1: unique tissue-specific functions revealed by selective gene knockout studies. Physiol. Rev..

[26] Calvo D, Gomez-Coronado D, Suarez Y, Lasuncion MA, Vega MA (1998). Human CD36 is a high affinity receptor for the native lipoproteins HDL, LDL, and VLDL. J. Lipid Res..

[27] Yu L (2002). Overexpression of ABCG5 and ABCG8 promotes biliary cholesterol secretion and reduces fractional absorption of dietary cholesterol. J. Clin. Invest..

[28] Ontsouka EC (2016). Can widely used cell type markers predict the suitability of immortalized or primary mammary epithelial cell models?. Biol. Res..

[29] Viturro E (2009). Cholesterol synthesis in the lactating cow: Induced expression of candidate genes. J. Steroid Biochem. Mol. Biol..

[30] Feingold KR, Moser AH (1985). Effect of lactation on cholesterol synthesis in rats. Am. J. Physiol..

[31] Bourlieu C, Michalski MC (2015). Structure-function relationship of the milk fat globule. Curr. Opin. Clin. Nutr. Metab. Care.

[32] Bitman J, Wood DL, Mehta NR, Hamosh P, Hamosh M (1986). Comparison of the cholesteryl ester composition of human milk from preterm and term mothers. J. Pediatr. Gastroenterol. Nutr..

[33] Jensen RG, Ferris AM, Lammi-Keefe CJ, Henderson RA (1990). Lipids of bovine and human milks: a comparison. J. Dairy Sci..

[34] Michalski MC, Briard V, Michel F, Tasson F, Poulain P (2005). Size distribution of fat globules in human colostrum, breast milk, and infant formula. J. Dairy Sci..

[35] Gallier S (2015). A novel infant milk formula concept: Mimicking the human milk fat globule structure. Colloids Surf. B Biointerfaces.

[36] Bayley TM (1998). Influence of formula versus breast milk on cholesterol synthesis rates in four-month-old infants. Pediatr. Res..

[37] Martinéz I (2013). Diet-induced alterations of host cholesterol metabolism are likely to affect the gut microbiota composition in hamsters. Appl. Environ. Microbiol..

[38] Midtvedt AC, Midtvedt T (1993). Conversion of cholesterol to coprostanol by the intestinal microflora during the first two years of human life. J. Pediatr. Gastroenterol. Nutr..

[39] Dimova LG, Zlatkov N, Verkade HJ, Uhlin BE, Tietge UJF (2017). High-cholesterol diet does not alter gut microbiota composition in mice. Nutr. Metab. (Lond).

[40] Ishibashi S (1993). Hypercholesterolemia in low density lipoprotein receptor knockout mice and its reversal by adenovirus-mediated gene delivery. J. Clin. Invest..

[41] Klett EL (2004). A mouse model of sitosterolemia: absence of Abcg8/sterolin-2 results in failure to secrete biliary cholesterol. BMC Med..

[42] Solca C, Tint GS, Patel SB (2013). Dietary xenosterols lead to infertility and loss of abdominal adipose tissue in sterolin-deficient mice. J. Lipid Res..

[43] Shipman LJ, Docherty AH, Knight CH, Wilde CJ (1987). Metabolic adaptations in mouse mammary gland during a normal lactation cycle and in extended lactation. Q. J. Exp. Physiol..

[44] Bligh EG, Dyer WJ (1959). A rapid method of total lipid extraction and purification. Can. J. Biochem. Physiol..

[45] Wiersma H (2009). Scavenger receptor class B type I mediates biliary cholesterol secretion independent of ATP-binding cassette transporter g5/g8 in mice. Hepatology.

[46] Dikkers A, Freak de Boer J, Annema W, Groen AK, Tietge UJ (2013). Scavenger receptor BI and ABCG5/G8 differentially impact biliary sterol secretion and reverse cholesterol transport in mice. Hepatology.

[47] Ichihara K, Fukubayashi Y (2010). Preparation of fatty acid methyl esters for gas-liquid chromatography. J. Lipid Res..

[48] Previs SF (2011). Quantifying cholesterol synthesis *in vivo* using (2)H(2)O: enabling back-to-back studies in the same subject. J. Lipid Res..

[49] Yao L, Dawson PA, Woollett LA (2003). Increases in biliary cholesterol-to-bile acid ratio in pregnant hamsters fed low and high levels of cholesterol. Am. J. Physiol. Gastrointest. Liver Physiol..

[50] Schonewille M (2016). Statins increase hepatic cholesterol synthesis and stimulate fecal cholesterol elimination in mice. J. Lipid Res..

